# Dosing of Bacterial Phytase (EC 3.1.3.26) in Laying Hens’ Diets

**DOI:** 10.3390/ani14223219

**Published:** 2024-11-09

**Authors:** Guilherme Souza Lima, Danilo Teixeira Cavalcante, Ricardo Romão Guerra, José Humberto Vilar da Silva, Alexandre Barbosa de Brito, Gilson Alexandre Gomes, Matheus Ramalho Lima, Marcos Aurélio Victor de Assunção, Carlos Henrique do Nascimento, Adiel Vieira de Lima, Paloma Eduarda Lopes de Souza, Fernando Perazzo Costa

**Affiliations:** 1 Animal Science Departament, Federal University of Paraiba, Campus Areia, Areia 58397-000, Paraiba, Brazil; guilherme_slima@hotmail.com (G.S.L.); danilo.cavalcante@ufape.edu.br (D.T.C.); rromaoguerra@gmail.com (R.R.G.); vilardasiva@yahoo.com.br (J.H.V.d.S.); henrike9@hotmail.com (C.H.d.N.); adiel1205@hotmail.com (A.V.d.L.); palomaeduardasbu01@gmail.com (P.E.L.d.S.); 2 Ab Vista, 3 Woodstock Court, Marlborough SN8 4AN, UK; alexandre.barbosa@abvista.com (A.B.d.B.); gilson.gomes@abvista.com (G.A.G.); 3 Animal Science Departament, Federal University of the Semi-Arid Region, Campus Mossoró, Mossoro 59625-900, Rio Grande do Norte, Brazil; marcos.assuncao@alunos.ufersa.edu.br

**Keywords:** phytase, commercial laying hens, supplementation, egg production

## Abstract

Despite the acknowledged advancements in the utilization of phytase dosing, limited research has been conducted on commercial laying hens. To assess the effectiveness of doses of exogenous phytase, a total of 320 laying hens aged between 44 and 64 weeks were utilized. These hens were divided into five treatments, which were then distributed using a completely randomized design with eight replicates, each containing eight birds. The treatments consisted of phytase supplementation levels of 0, 500, 1000, 1500, and 3000 FTU/kg. Quadratic regression equations were utilized to determine the maximum and minimum values for the levels of phytase supplementation. A significant difference was observed in the levels of phytase supplementation, where a quadratic model was fitted to various variables including egg weight (g), egg mass (g/bird/day), feed conversion per egg mass (g/g), yolk concentration (%), albumen concentration (%), shell thickness (μm), shell strength (kgf), specific gravity, and yolk color. Further, an increased supplementation improved the digestive tract of the hens, thereby providing a better villus–crypt ratio. Considering the advantageous effects of phytase dosing, it is recommended to supplement corn and soybean meal-based diets with 1500 FTU/kg of bacterial phytase for laying hens during the 44- to 64-week production period.

## Simple Summary

Laying hens possess a considerable capacity for digestion, yet certain foods contain compounds that hinder the function of digestive enzymes in avian species. These compounds, referred to as antinutritional factors, include phytate. Phytate degradation is facilitated by phytase. Through dosing, the complete elimination of phytate can be achieved, thereby enabling birds to optimize their utilization of food and nutrients previously associated with undigested compost. Consequently, the determination of the optimal phytase dosage for phytate degradation in diets for laying hens during the egg production phase assumes paramount importance. Therefore, the objective of this study was to evaluate this aspect, taking into consideration various physiological and productive efficiency metrics related to laying hens.

## 1. Introduction

The utilization of exogenous enzymes in poultry diets has emerged as a strategy to enhance animal production and minimize production expenses. Additionally, it has the potential to counteract the antinutritional repercussions that certain feed ingredients may have on the animals. Furthermore, the incorporation of these exogenous enzymes has resulted in improved ingredient utilization, facilitating their digestion and metabolic processes in birds, while simultaneously reducing the excretion of environmentally detrimental substances.

The enzymes utilized include phytase, which functions by catalyzing and hydrolyzing phytate molecules, typically yielding inositol, inositol monophosphate, and inorganic phosphate. This process enhances the availability of phosphorus for absorption [1]. Nevertheless, it has been observed that phytate can have detrimental effects on the digestion and absorption of other minerals, amino acids, and energy [2]. Furthermore, it acts as an intestinal irritant, leading to increased endogenous losses [3]. Consequently, new concepts regarding the utilization of this enzyme have emerged as further studies are conducted [4]. This is because the extra-phosphoric effects of phytases enable their application for purposes beyond the liberation of phosphorus from phytic acid molecules [5].

The phytase enzyme demonstrates additional phosphoric activity during animal digestion. These enzymes enhance the digestibility of minerals, amino acids, and energy when incorporated into the diet. Additionally, they contribute to the function of essential endogenous enzymes in metabolism and promote elevated concentrations of hepatic carotenoid, α-tocopherol, retinol, and coenzyme Q10 [6]. Moreover, this enzyme can impact the insulin receptor sensitivity of the chicken liver and inhibit the expression of the insulin-like growth factor-1 gene [7]. Considering these metabolic benefits, researchers have explored the concept of phytase dosing to determine the optimal level of enzyme supplementation in poultry diets.

Despite the well-documented advancements in the utilization of phytase dosing, there is a lack of research conducted on commercial laying hens. This study aims to address this gap by investigating the impact of dietary supplementation with doses of exogenous phytase on laying hens in their second production cycle (44 to 64 weeks). The hypothesis behind this study is that phytase hydrolyzes phytic acid molecules, thereby enhancing the digestibility and availability of essential minerals, amino acids, and energy. Additionally, the study examines the potential effects on the development of the digestive tract, as well as the improvements in the productive performance of laying hens.

## 2. Materials and Methods

The experimental protocol was approved by the Animal Research Ethics Committee of the Federal University of Paraiba, Brazil, by certificate of approval no. 023/2017.

### 2.1. Experimental Design, Facilities, and Diets

The experiment was conducted at the Poultry Module of the Department of Animal Science at the Center of Agricultural Sciences, UFPB, Campus II, located in Areia, Paraiba (PB), Brazil. Throughout the experiment, temperature and relative humidity were recorded daily, yielding a mean temperature of 26 °C and an average relative humidity of 87% during the entire experiment period.

A total of 320 Hy-line W-36 laying hens, aged 44 to 64 weeks, were utilized for a duration of 20 weeks, starting with a mean initial productivity of 94%. The experimental design consisted of five treatments arranged in a completely randomized design, with eight replicates of eight hens each.

Quantum Blue 5000 (AB Vista, Marlborough, Wiltshire, UK) is indeed a commercial formulation of phytase that falls under the classification of 6-phytase (EC 3.1.3.26). It is derived from *Escherichia coli* and is designed to enhance the digestibility of phytate in animal feeds, thereby improving phosphorus availability for laying hens. In our study, we utilized Quantum Blue 5000 as the source of phytase to evaluate its efficacy at varying inclusion levels (0, 500, 1000, 1500, and 3000 FTU/kg) in layer diets.

To ensure consistency in our manuscript, we will refer to Quantum Blue 5000 as “6-phytase (EC 3.1.3.26) derived from Quantum Blue 5000” in all relevant sections.

The hens were housed in a conventional laying house with clay tile roofing, equipped with feeding troughs and nipple drinkers. They were grouped in galvanized wire cages measuring 45 cm × 45 cm × 30 cm, where they had ad libitum access to water and feed. The lighting schedule followed the recommendations from the Hy-line W36 breeding manual, providing 16 h of light per day—12 h of natural light and 4 h of continuous artificial light from fluorescent lamps.

The diets were meticulously formulated to meet the nutritional requirements specified in the Hy-line W36 breeding manual, ensuring that they were both isoproteic and isoenergetic. To assess the effects of phytase levels, the diets included 0, 500, 1000, 1500, and 3000 FTU/kg of phytase, with inert (washed sand) ingredients being replaced by corresponding doses of the enzyme in the proportions of 0, 0.100, 0.200, 0.300, and 0.600 kg/ton (Table 1). This approach allows for a direct comparison of phytase efficacy and its influence on nutrient absorption rates in laying hens, emphasizing the need for a tailored phytase application based on specific feed formulations and processing technologies.

### 2.2. Performance Analysis

In the 15 days prior to the start of the experiment, the production of the birds was recorded and used as the basis for the distribution and homogenization of the experimental treatments.

The experimental period was divided into 5 subperiods of 28 days. Egg collection was performed daily in the afternoon after the production of each plot was recorded; in the case of mortality, corrections were made as needed. In the last three days of each subperiod, all eggs were weighed, and six eggs per plot were selected based on the mean weight. Of these, three eggs were designated for egg quality analysis, two for specific gravity analysis, and one for shell strength analysis. This selection method was chosen because it is widely applied in research involving laying hens. If all data were analyzed from the same eggs, factors such as the presence of water during the specific gravity measurement could influence internal quality parameters, including the Haugh unit. Additionally, there was no target weight for egg selection; instead, the choice was made based on the average weight to ensure consistency in the analyses. Consequently, a total of 720 eggs were analyzed per treatment at the end of the experiment (6 eggs × 3 days × 8 replicates × 5 subperiods).

The analyzed variables related to performance and egg quality were as follows: feed intake (g/bird/day); productivity (%); egg weight (g); egg mass (g/bird/day); feed conversion per egg mass (kg/kg) and per dozen eggs (kg/dz); percentages (%) of yolk, albumen, and shell; shell thickness (μm); Haugh unit; yolk color; specific gravity (g/cm3); shell strength (kgf); and viability (%).

The yolk color was analyzed using a DSM color fan (DSM Nutritional Products, Kaiseraugst, Switzerland), following the recommended methodology. To ensure scoring consistency, all measurements were taken by the same trained personnel, and blind parallel scoring was performed to validate the results.

### 2.3. Intestinal Morphometry

At the end of the experiment, one bird per replicate was chosen based on the mean weight of the plot and then euthanized for the subsequent collection of biological material. A 1 cm fragment was collected from the middle portion of the duodenum of each bird, with each treatment comprising 8 fragments; these fragments were fixed by immersion in 10% formaldehyde. The tissue fragments were embedded in paraffin according to standard histological procedures [8]. Next, 5 µm sections were cut from each paraffin block in a microtome, and the histological slides were stained with “periodic acid–Schiff” (PAS) and scanned with a Motic camera (Motic Instruments Inc., Xiamen, China) coupled to an Olympus BX-53 microscope (Olympus Corporation, Tokyo, Japan) with Motic Image Plus 2.0 image analyzer software (Motic Instruments Inc., Xiamen, China).

For each photomicrograph, three measurements of the intestinal villus height and its respective crypt were taken, totaling 72 measurements (8 animals × 3 photomicrographs × 3 measurements) for each variable mentioned above per treatment. The villus height (VH, μm) was measured from the region of the intestinal mucosa that coincided with the upper portion of the crypts until its apex. The crypt depth (CD, μm) was the distance between the villus base to the crypt–villus transition region. The villus–crypt (V:C) ratio was determined by the ratio of the villus height to the crypt depth. The histomorphometric analyses were performed by a single histologist to avoid interpretation errors.

### 2.4. Glycogen and Hepatic Steatosis

This analysis adopted the same standard histological and image capture procedure that was described for intestinal morphometry. A 0.5 cm^3^ fragment of the left lateral lobe of the liver of each bird was collected, with each treatment comprising 8 fragments. For the assessment of the glycogen levels and grade of hepatic steatosis, an evaluation score was assigned to each liver analyzed using liver photomicrographs of each animal. Histological slides were also stained with PAS.

Four liver photomicrographs were used for the evaluation of the glycogen concentration and hepatic steatosis score for each animal, totaling 32 samples (8 animals × 4 photomicrographs), which were analyzed by assigning a score for the glycogen concentration and grade of steatosis. For the glycogen concentration, scores were assigned according to the presence of glycogen in the liver of the birds: 0 (no positivity), 1 (little positivity), 2 (moderate positivity), and 3 (intense positivity). Scores for the grade of hepatic steatosis were defined considering the amount and size of the cytoplasmic lipid vacuoles in hepatocytes in PAS staining as follows: 0 (no steatosis), 1 (mild steatosis), 2 (moderate steatosis), and 3 (advanced steatosis) in a modified version of Ishak’s semiquantitative scoring system [9].

### 2.5. Morphometry of the Magnum

The standard histological and image capture procedure described above was used for this analysis. A 1 cm fragment from the middle and thicker region of the magnum was collected per animal, with each treatment comprising 8 fragments. Histological slides were also stained with PAS. Two photomicrographs were used for each animal for the morphometric assessment of the magnum, totaling 16 samples (8 animals × 2 photomicrographs). In this material, the folds of the magnum, maturation of the glands, and the thickness of the epithelium of the magnum were evaluated. To measure the epithelium of the magnum, a measurement was taken from each scanned photomicrograph, totaling 16 measurements per treatment.

### 2.6. Uterine Folds

The standard histological and image capture procedure described above was used for this analysis. A 1 cm^3^ fragment was collected from the middle region of the uterus per animal, with each treatment comprising 8 fragments. Histological slides were also stained with PAS, and two photomicrographs were scanned per animal, for 16 samples (8 animals × 2 photomicrographs), which were analyzed by assigning a score for the degree of branching of the folds of the uterus, as follows: 0 (few secondary folds), 1 (moderate secondary folds), 2 (intense secondary folds), 3 (moderate secondary and tertiary folds), and 4 (intense secondary folds and tertiary folds), in a modified version of Ishak’s semiquantitative scoring system [9].

### 2.7. Statistical Analysis

Statistical software R version 3.5.1 was used for the statistical analysis of the variables for performance, egg quality, and morphometry of the digestive tract (duodenum) and reproductive system of laying hens (magnum). An analysis of variance was performed, and regression analysis at 5% probability was used for the effects of the FTU levels. When significant, the equations were presented, and quadratic regression equations were derived for estimating the maximum and minimum values of the levels of phytase supplementation.

Statistical software R version 3.5.1 was used for the analysis of the liver glycogen score, hepatic steatosis, and uterine folds. For this purpose, a multivariate ordinal logistic regression analysis was performed using a cumulative logit model to estimate the relative probabilities of the scores adapted for the variables within the FTU supplementation levels in the diets modeled by the multinomial distribution of the data. The ordinal regression techniques model the cumulative probabilities that a given response (Y) falls in or below a given category (j) for all possible values of j [10]. When significant, graphs were generated for visualization of the behavior of the data.

## 3. Results

### 3.1. Performance and Egg Quality

Table 2 presents the outcomes for performance and egg quality variables of laying hens aged 44 to 64 weeks. These hens were fed doses of bacterial phytase (EC 3.1.3.26). Our analysis revealed a significant impact of the treatments on the following variables: egg weight (*p* = 0.0166), egg mass (*p* = 0.0056), feed conversion per egg mass (*p* = 0.0168), % yolk (*p* = 0.0442), % albumen (*p* = 0.0078), yolk color (*p* = 0.0502), specific gravity (*p* < 0.0001), and shell strength (*p* = 0.0249). In all cases, the regression analysis supported a quadratic model, and the levels of phytase supplementation were estimated by deriving the corresponding equations. Regarding egg weight, supplementing the diets with 1643 FTU/kg would have resulted in heavier eggs, while a larger egg mass would be achieved with a supplementation of 1450 FTU/kg. Furthermore, a supplementation level of 1250 FTU/kg would have improved the feed conversion per egg mass. For yolk and albumen concentrations, the maximum values were estimated at 1333 and 1500 FTU/kg, respectively. Additionally, for shell thickness and shell strength, the optimal levels of phytase supplementation were projected to be 1217 FTU/kg and 750 FTU/kg, respectively. Notably, a supplementation of 1750 FTU/kg would enhance the specific gravity of the eggs, whereas 1875 FTU/kg of phytase supplementation would result in eggs with a darker yolk color.

No significant differences were observed between treatments with varying levels of phytase supplementation for the following variables: feed intake (*p* = 0.2062), productivity (*p* = 0.3793), feed conversion per dozen eggs (*p* = 0.5733), percentage of shell (P = 0.6658), Haugh unit (*p* = 0.5965), and viability (*p* = 0.8291) (Table 2).

### 3.2. Intestinal Morphometry and Reproductive System

Table 3 presents the data from the morphometric analyses of the duodenum of laying hens, including measurements of the crypt depth (CD, μm), villus height (VH, μm), villus–crypt ratio (V:C, μm), and epithelium of the magnum (EPM, μm).

A quadratic effect was observed for the levels of FTU supplementation on the duodenal morphometric variables of laying hens in the second production cycle (*p* < 0.0001). A supplementation level of 1253 FTU/kg is estimated to be sufficient for achieving a lower CD (μm). For a higher V:C (μm) ratio, the ideal supplementation level is 1888 FTU/kg.

**Figure 1:**
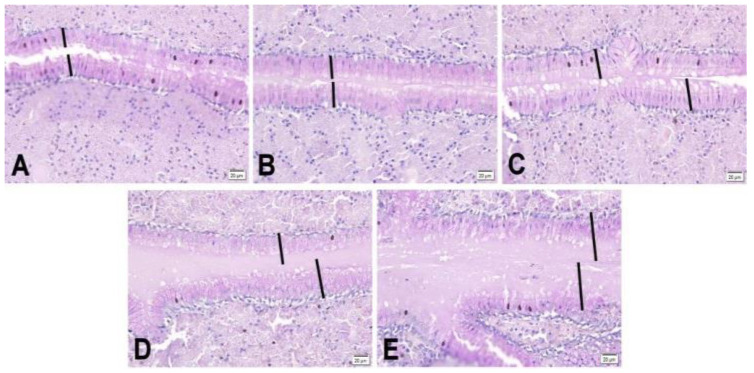
Photomicrographs of the epithelium of the magnum of 64-week-old laying hens fed different levels of FTU in the diets. (**A**) 0 FTU; (**B**) 500 FTU; (**C**) 1000 FTU; (**D**) 1500 FTU; (**E**) 3000 FTU. The black bars show the thickness of the epithelium of the magnum in the different treatments. Periodic acid–Schiff staining.

No morphological differences were observed in the folds of the magnum among the different treatments, as the maturation stage of its glands did not differ. However, changes in the thickness of the magnum epithelium were observed (Figure 1). A linear trend was observed for the effect of phytase supplementation levels on the morphometric variable of the magnum’s epithelium, with higher levels of FTU supplementation corresponding to a higher EPM (μm).

Figure 1 shows the thickness of the epithelium of the laying hens that were fed doses of the phytase in the second production cycle. The higher the FTU level was in the diets, the greater the thickness of the magnum.

**Figure 2:**
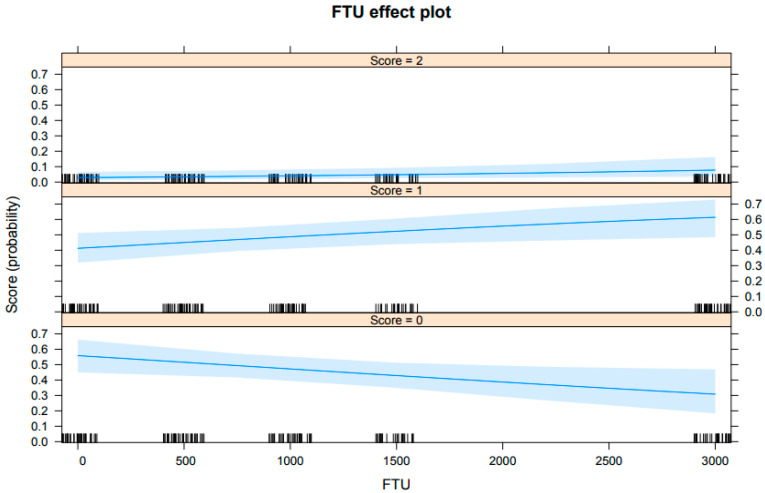
Hepatic steatosis score of 64-week-old laying hens fed different levels of FTU in the diets. Score: 0 (no steatosis), 1 (mild steatosis), 2 (moderate steatosis), and 3 (advanced steatosis).

In relation to the hepatic steatosis score variable, an inverse correlation was observed between the occurrence of a steatosis score of 0 (indicating no presence of steatosis) and the levels of FTU in the diet. Specifically, as the levels of phytase increased, the birds exhibited an emergence of cytoplasmic lipid vacuoles in their hepatocytes. Furthermore, it was found that a higher probability of the occurrence of grade 1 steatosis, characterized by mild steatosis, was associated with higher FTU levels. Notably, no instances of advanced steatosis (score 3) were reported for the animals (refer to Figure 2).

**Figure 3:**
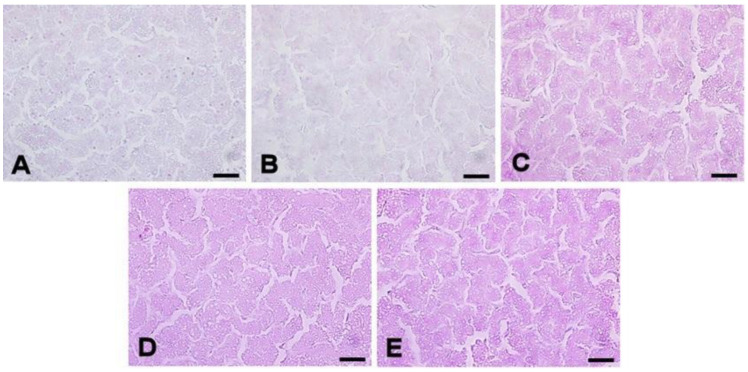
Photomicrographs of the liver of 64-week-old laying hens fed different levels of FTU in the diets. (**A**) 0 FTU; (**B**) 500 FTU; (**C**) 1000 FTU; (**D**) 1500 FTU; (**E**) 3000 FTU. Periodic acid–Schiff staining.

The presence of a larger number of cytoplasmic lipid vacuoles in the hepatocytes, characterized by a score of 1, was more evident in the animals fed 1500 FTU in the diets (Figure 3).

**Figure 4:**
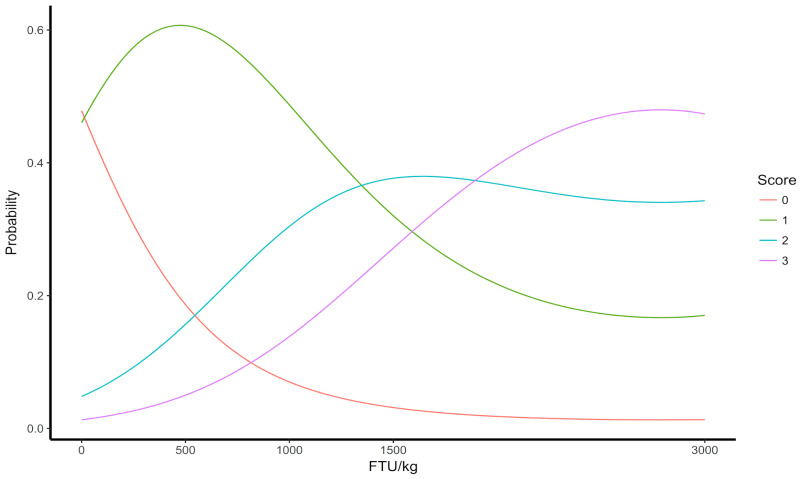
Liver glycogen concentration in 64-week-old laying hens fed different levels of FTU in the diets. *0 (no positivity), 1 (little positivity), 2 (moderate positivity), and 3 (intense positivity).

Figure 4 depicts the analysis of the liver glycogen concentration in laying hens fed elevated doses of bacterial phytase. The graph illustrates a linear trend in the results, indicating that as the FTU levels in the experimental diets increased, there was a corresponding increase in glycogen concentration. Consequently, the likelihood of observing scores 0 and 1, which indicate absence and low glycogen positivity, respectively, decreased.

**Figure 5:**
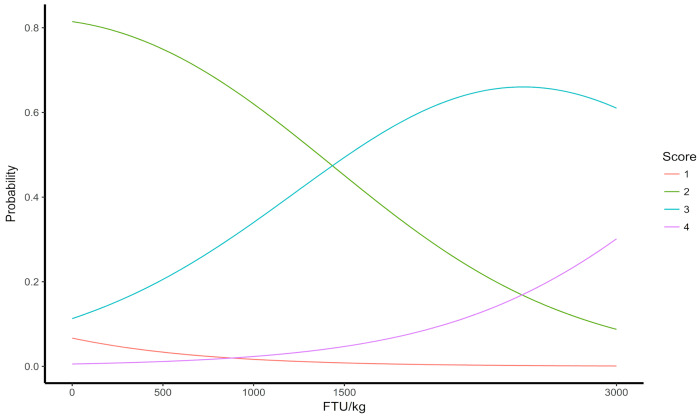
Evaluation of the score of uterine folds of 64-week-old laying hens fed different levels of FTU in the diets. Few folds—1, moderate secondary folds—2, moderate secondary and tertiary folds—3, and intense tertiary folds—4.

Figure 5 exhibits a linear relationship between the levels of phytase supplementation and the evaluation scores of uterine folds in the reproductive system of laying hens during the second production cycle. The absence of phytase supplementation in the diets was linked to a higher likelihood of regular secondary folds (score 2) occurring. As the levels of FTU increased in the diets, this probability decreased.

Supplementing the diets with high doses of phytase, specifically 1500 and 3000 FTU/kg, resulted in an increase in the occurrence of moderate secondary and tertiary folds (score 3), as well as intense tertiary folds (score 4) in the birds’ uterus.

**Figure 6:**
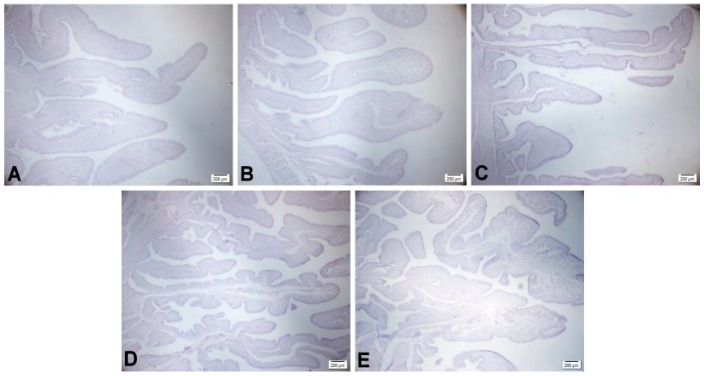
Photomicrographs of the uterine folds of 64-week-old laying hens fed different levels of FTU in the diets. (**A**) 0 FTU; (**B**) 500 FTU; (**C**) 1000 FTU; (**D**) 1500 FTU; (**E**) 3000 FTU. Periodic acid–Schiff staining.

The analysis of the photomicrographs of the uterus of the laying hens (Figure 6) provided further clarification for the results obtained in the statistical analysis conducted on the scores of the uterine folds. Image “A” (T1—0 FTU) clearly illustrates the number of folds present in the uterus of these animals. Moreover, there is a noticeable increase in the number of folds as depicted in image “B” (T2—500 FTU) and image “C” (T3—1000 FTU). Images “D” (T4—1500 FTU) and “E” (T5—3000 FTU) correspond to scores of 3 and 4, respectively, indicating a higher number of uterine folds.

## 4. Discussion

The aim of this study was to assess the effect of supplementing the diet of laying hens during the second production cycle (44 to 64 weeks) with exogenous phytase dosing. The underlying hypothesis for this investigation was that phytase facilitates the hydrolysis of phytic acid molecules, thereby enhancing the digestibility and bioavailability of minerals, amino acids, and energy. Furthermore, it was postulated that phytase supplementation contributes to the development of the birds’ digestive tract and positively impacts the reproductive system of laying hens, ultimately improving their overall productive performance.

The utilization of phytase as a supplement in poultry diets is intended to liberate phosphorus from phytate molecules, as well as counteract the negative effects on nutrient digestion and absorption caused by the presence of phytic acid. To achieve these supplementary effects, it is recommended to administer a high dosage of phytase. On average, a supplementation level of 1500 FTU/kg in diets comprised of maize and soybean meal has been shown to enhance the performance and egg quality of hens during the second production cycle.

Several factors analyzed in this study elucidate the mechanisms through which the excessive administration of phytase can positively influence the zootechnical performance of laying hens. One crucial aspect that may impact animal performance and nutrient utilization is the intestinal morphometry of the animals [11]. The findings of the present investigation reveal a higher villus–crypt ratio, suggesting an enhancement in the digestive tract of the hens due to phytase dosing. This improvement in gut health is likely attributed to the inhibition of phytate activity.

In the study conducted by Güler et al. [12], the observation that a supplementation of bacterial phytase (EC 3.1.3.26) in layer diets enhances intestinal morphology—specifically through an increased villi height—can be elucidated through two primary mechanisms. Firstly, the metabolic effects of the phosphorus (P) released from phytic acid may positively influence the health of intestinal epithelial cells and enhance nutrient absorption, thereby contributing to the development of healthier villi. Secondly, the inert ingredients in the diet can modify feed characteristics, promoting beneficial fermentation processes within the gastrointestinal tract. This altered environment may support the proliferation of advantageous microbiota, further stimulating the development of intestinal structures. Collectively, the observed improvements in intestinal morphology are likely attributable to both the metabolic benefits associated with phosphorus release and the physiological changes induced by the inert ingredients, thereby providing a comprehensive understanding of phytase’s role in layer nutrition.

A higher ratio of villi to crypts, which is characterized by shorter crypts and longer villi, is indicative of reduced cell proliferation. This decrease in protein turnover in the intestinal epithelium results in a decrease in the animal’s maintenance energy requirements, enabling energy allocation for production and serving as an indicator of improved intestinal health. This histomorphometry variable is directly associated with production data as it plays a crucial role in the digestion and absorption of nutrients necessary for the animals’ energy metabolism [13]. This, in turn, is reflected in the production and egg quality of laying hens.

Animals that receive an average supplementation of 1500 FTU/kg in their diets exhibit enhanced yolk and albumen production, leading to an increased final egg weight. These findings hold significance in the analysis of productive efficiency, as they indicate that the animals do not need to consume larger quantities of feed in order to produce heavier eggs. The incorporation of phytase into the diets offers this advantageous effect, resulting in an improved feed conversion per egg mass.

The increase in egg weight resulting from the addition of exogenous enzymes can be attributed to the hydrolysis of phytate. When phytate is present in the diet, it forms complexes with nutrients, rendering phosphorus unavailable and potentially interfering with the availability of amino acids. This can have a detrimental effect on animal performance, as phytate acts as an antinutritional factor [14]. However, the inclusion of phytase in excessive amounts leads to the efficient breakdown of phytic acid molecules. This is evidenced by the enhanced productive performance of the birds as phytase supplementation levels in the diets increase. Therefore, the activity of the enzyme is constrained by the presence of the substrate (phytate). This explains the quadratic effect observed for the variables being discussed, as the enzyme activity is limited to the quantity of phytic acid present in the diets.

The mechanism of action of the enzyme, which catalyzes the release of phosphorus from the phytate molecule, has been elucidated in previous studies [15,16,17]. This mechanism leads to an increased concentration of circulating phosphorus in the animal’s body. The enhanced availability of this mineral directly impacts the production of eggs in laying hens, as phosphorus is involved in the synthesis of albumin found in the egg white and is also stored in the yolk as phospholipids and phosphoprotein. This process is supported by the higher concentrations of albumen and yolk observed in the eggs of hens supplemented with phytase.

The observations regarding albumen deposition in eggs reveal an interesting relationship between phytase supplementation and the physiological changes within the magnum. Although no significant differences were found in the folds of the magnum or the number of more active albumen cells across treatments, the linear increase in the thickness of the magnum epithelium suggests that higher levels of phytase may enhance the efficiency of the egg formation process.

The thickening of the epithelium can be linked to several factors. Phytase supplementation may improve the availability of phosphorus and other nutrients, which could stimulate epithelial cell proliferation and differentiation. A thicker magnum epithelium is likely to produce more mucus, which plays a crucial role in the albumen deposition process. This mucus facilitates the movement of albumen through the magnum, potentially reducing the time an egg spends in this section of the oviduct and thus accelerating overall egg production. Furthermore, the increased ciliation of the epithelium associated with its thickness can enhance the transportation of albumen, as ciliated cells help propel the egg white along the oviduct. This mechanical action, combined with the increased mucus production, could lead to a more efficient deposition of albumen, contributing to the observed outcomes. In summary, while the direct metabolic pathways remain speculative, it can be inferred that phytase supplementation enhances the physiological responses within the magnum, leading to improved albumen production efficiency and quicker egg formation processes, as supported by the findings in [18].

Another analyzed variable that is influenced by a higher deposition of egg components, particularly the yolk, is the presence of hepatic steatosis. The appearance of cytoplasmic lipid vacuoles in the hepatocytes is directly linked to the synthesis of estrogen, a hormone that plays a crucial role in avian ovulation [19]. In this context, a steatosis score of 1 was more likely to occur when the birds were fed diets containing 1500 FTU. It has been suggested that the increased fat content in the liver may also be attributed to the heightened synthesis of estrogen in the ovary, which occurs at the expense of high rates of egg production in this phase [20]. During the egg laying phase, when all organs and systems are fully developed, the surplus of liver glycogen, previously utilized as an energy substrate during growth phases, starts to accumulate in the liver. It is then used for enhanced estrogen production, ultimately leading to an increase in egg production.

Some challenges related to the production of a higher proportion of egg yolk and albumen may arise in the context of eggshell formation and breaking strength. However, the inclusion of phytase in the diets of laying hens has been found to result in eggs with thicker and stronger shells. The calcification process of the eggshell is closely linked to calcium and phosphorus metabolism and is influenced by the development of the uterus, which serves as the location for the entire eggshell formation process [21].

In this particular study, the treatments applied to the laying hens were observed to have a significant impact on the histological structure of the uterus. These findings are consistent with previous research that has established a connection between eggshell thickness and strength. Specifically, it was determined that diets containing approximately 1217 and 750 FTU/kg of phytase, for corn-based and soybean meal-based diets, respectively, would yield eggs with thicker and stronger shells. An examination of the uterine folds revealed that as the level of phytase supplementation increased, this reproductive compartment underwent greater development. In other words, higher levels of FTU in the diets were associated with an increased number of uterine folds.

The presence of uterine folds demonstrates the uterus’s ability to deposit calcium carbonate during eggshell formation, thereby expediting the process. Hence, the administration of an excessive amount of phytase in the diets of laying hens promotes the enhanced development of the uterus, leading to a more efficient deposition of calcium salts in the eggshell. As a result, the eggshell becomes thicker and, consequently, stronger.

In addition to its effects on eggshell thickness, the overdosing of phytase also influences the specific gravity of the eggs, with a quadratic relationship between the levels of supplementation. The inclusion of 1750 FTU/kg of phytase in the diets was found to result in eggs with a higher specific gravity. It is worth noting that the specific gravity of eggs is directly influenced by the thickness of the eggshell [22], meaning that as eggshell thickness increases, so does the specific gravity. Thus, the overdosing of phytase has a direct impact on the deposition of calcium in the eggshells, resulting in increased shell thickness and improved egg quality. The yolk color was influenced by the addition of phytase, and a quadratic effect was observed for this parameter. Consequently, it is predicted that the inclusion of 1875 FTU/kg of phytase in the diets would result in eggs with darker yolks. The color of the yolk is attributed to the presence of carotenoids in the bird’s diet. Since these animals are unable to synthesize these compounds, they must be obtained through dietary absorption [23,24]. In a study conducted by [6], higher levels of carotenoids were found in the liver of broilers fed increasing amounts of phytase. These findings may explain the darker egg colors observed in the present study following supplementation with high doses of phytase. It is plausible that a greater absorption of carotenoids occurred, which were then stored in the liver and subsequently transferred to the ovary, ultimately influencing yolk color.

In addition to affecting yolk color, it is evident that dosing with phytase has a substantial impact on the overall energy metabolism of the bird. Enhanced phytate hydrolysis can improve animal performance and enhance nutrient utilization efficiency, thereby increasing production efficiency for poultry farmers [25]. The present study corroborates these findings, highlighting improvements in the energy metabolism of the bird when evaluating liver glycogen reserves, with higher reserves observed when phytase supplementation levels were increased. Liver glycogen serves as the primary energy reserve for animals and is primarily utilized to maintain glucose homeostasis. However, in order for glucose to be stored in the liver, metabolic processes must be balanced. The presence of phytate in the diet disrupts this balance, particularly impacting sodium excretion [14]. Given the strong relationship between phytate and metabolism, it is imperative to neutralize the effects of phytate and reduce sodium excretion.

One of the detrimental consequences of increased sodium excretion is the impairment of the sodium and potassium pump’s functionality. A study by [26] demonstrated a decrease in sodium pump activity when animals were fed a diet containing phytic acid, as evidenced by measurements of blood glucose levels. Conversely, a study by [27] showed an increase in sodium pump and glucose concentrations in the enterocytes of the duodenum and jejunum in chickens fed diets supplemented with exogenous phytase. Accordingly, ref. [28] proposed that the presence of phytate hampers glucose absorption by affecting the functionality of the sodium and potassium pump. Phytase was found to enhance sodium (Na) retention and significantly improve the digestibility of 16 amino acids in the proximal jejunum of avian species [29]. The authors proposed that increased Na retention could positively impact amino acid digestion and glucose absorption, and this hypothesis was supported by [30], which confirmed higher sodium reabsorption in the small intestine when phytase was included in the diets. This subsequent increase in available sodium facilitated nutrient co-transport through the sodium pump.

Consequently, the administration of a higher dosage of phytase resulted in enhanced glucose absorption by the animals, which was then stored as liver glycogen. The accumulation of glycogen suggests a heightened effectiveness of the sodium and potassium pump in erythrocytes. However, due to the lack of evaluation regarding the functionality of the sodium and potassium pump, as well as sodium excretion, further investigations are necessary to establish a definitive connection between phytase dosing and increased liver glycogen reserves in laying hens during their second production cycle.

Additionally, phosphorus plays a critical role in metabolic pathways that facilitate energy production, particularly through its involvement in adenosine triphosphate (ATP) synthesis. Adequate phosphorus levels are essential for supporting energy metabolism, which in turn influences the energy-to-protein ratios within the diet. When energy metabolism is optimized, it enables more efficient protein turnover and fulfills maintenance requirements, potentially enhancing growth and egg production. Therefore, a comprehensive understanding of the interplay between phosphorus metabolism and energy utilization is crucial for elucidating how phytase supplementation can contribute to overall nutrient efficiency and animal performance. Further research is warranted to investigate these metabolic pathways and their implications for the nutritional strategies employed for laying hens, particularly in relation to optimizing protein turnover and fulfilling the maintenance needs of the birds during their second production cycle.

## 5. Conclusions

For optimal performance and egg quality, it is advisable to provide laying hens with diets based on corn and soybean meal supplemented with 1500 FTU/kg of bacterial phytase during the 44 to 64 weeks of their production cycle. However, it is important to note that certain parameters were optimized at lower levels of phytase supplementation. This observation suggests that while 1500 FTU/kg can effectively enhance egg productivity and quality, lower phytase levels may also fulfill specific objectives related to the maintenance of the hens’ physical and physiological status. Therefore, future research should investigate the balance between phytase levels to achieve varying production goals while optimizing the overall health and welfare of laying hens throughout their production cycle.

## Figures and Tables

**Table 1:** Experimental diets with dosing of bacterial phytase for laying hens in the second production cycle (44 to 64 weeks).

Ingredients, g/kg	Treatment (Phytase—FTU Level)				
	0	500	1000	1500	3000
Corn, 7.88%			586.890		
Soybean Meal, 45.22%			249.390		
Soybean Oil			30.000		
Limestone			103.820		
Dicalcium phosphate 18.5%			19.940		
Salt			2.430		
DL-Methionine			2.120		
L-Lysine HCl			0.350		
L-Valine			0.050		
Na Bicarbonate			2.840		
Choline Chloride			0.700		
Mineral Premix ^1^			0.500		
Vitamin Premix ^2^			0.250		
Antioxidant ^3^			0.100		
Colistin			0.020		
Inert—Washed Sand ^4^	0.600	0.500	0.400	0.300	0.000
Phytase ^5^	0.000	0.100	0.200	0.300	0.600
Total (ton)	1.000	1.000	1.000	1.000	1.000
**Nutritional composition** (Calculated)					
FTU/kg	0	500	1000	1500	3000
Metabolizable Energy, kcal/kg			2822.000		
Crude Protein, %			16.060		
Met + Cys dig, %			0.660		
Met dig, %			0.433		
Lysine dig, %			0.780		
Threonine dig, %			0.550		
Valine dig, %			0.690		
Ca, %			4.480		
P avail, %			0.490		
Cl, %			0.210		
Na, %			0.190		
K, %			0.627		
Phytic Acid, %			0.198		
Electrolytic balance, mEq/kg			183.650		

^1^ Minimum premix per kilogram of feed: Mn: 60 g; Fe: 80 g; Zn: 50 g; Cu: 10 g; Co: 2 g; I: 1 g; Se: 250 mg. ^2^ Vitamin premix per kilogram of feed: vitamin A: 15,000,000 IU; vitamin D3: 1,500,000 IU; vitamin E: 15,000 IU; vitamin B1: 2.0 g; vitamin B2: 4.0 g; vitamin B6: 3.0 g; vitamin B12: 0.015 g; nicotinic acid: 25 g; pantothenic acid: 10 g; vitamin K3: 3.0 g; folic acid: 1.0 g; ^3^ Ethoxyquin; ^4^ washed sand; ^5^ Quantum Blue 5000; contribution of the enzyme supplementing 500 FTU: available phosphorus 0.150%, calcium 0.165%, sodium 0.035%, lysine 0.017%, methionine 0.004%, cystine 0.035%, threonine 0.033%, crude protein 0.421%, metabolizable energy 52 kcal/kg.

**Table 2:** Performance and egg quality of laying hens in the second production cycle when fed diets containing doses of phytase.

Treatment FTU Level	0	500	1000	1500	3000	CV (%)	*p*-Values	Regression	
								L	Q
Feed intake (g/bird/day)	107.91	108.44	106.34	107.03	106.89	1.76	0.2062	0.1725	0.319
Productivity (%)	89.43	90.07	89.99	89.39	87.75	2.84	0.3793	0.0912	0.3114
Egg weight (g) ^1^	64.17	65.19	65.87	66.03	64.97	1.50	0.0166	0.2506	0.0023
Egg mass (g/bird/day) ^2^	57.37	58.70	59.12	59.57	56.98	2.31	0.0056	0.2396	0.0005
Conversion per egg mass (kg/kg) ^3^	1.89	1.86	1.80	1.81	1.87	2.84	0.0168	0.8243	0.0013
Conversion per dozen eggs (kg/dz)	1.45	1.44	1.43	1.43	1.46	3.00	0.5733	0.4729	0.1676
Yolk (%) ^4^	26.44	26.20	25.89	25.97	26.61	2.15	0.0442	0.3407	0.0072
Albumen (%) ^5^	64.17	64.36	64.82	64.74	64.01	1.02	0.0809	0.481	0.0078
Shell (%)	9.36	9.39	9.27	9.27	9.35	2.18	0.6658	0.7425	0.2552
Shell thickness (μm) ^6^	385.33	389.61	391.54	388.64	380.79	1.94	0.0076	0.1077	0.0311
Shell strength (kgf) ^7^	3.19	3.39	3.36	3.3	2.71	10.69	0.0249	0.0061	0.0296
Yolk color ^8^	6.66	6.78	6.90	6.81	6.71	2.43	0.0502	0.9823	0.0059
Specific gravity ^9^	1.080	1.083	1.086	1.089	1.080	0.32	<0.0001	0.8956	<0.0001
Haugh unit	81.37	80.89	81.12	81.17	80.26	1.78	0.5965	0.1628	0.6484
Viability (%)	98.43	98.21	100	98.21	97.65	4.16	0.8291	0.6316	0.5733

Regression equation to individual variable: ^1^ Egg weight: y = −0.0000007x^2^ + 0.0023x + 64.2; R^2^ 0.99; Max: 1643 FTU/kg; ^2^ Egg mass: y = −0.000001x^2^ + 0.0029x + 57.399; R^2^ 0.99; Max 1450 FTU/kg; ^3^ Conversion per egg mass: y = 0.00000004x^2^ + 0.0001x + 1.8968; R^2^ 0.94; Min 1250 FTU/kg; ^4^ % Yolk: y = 0.0000003x^2^ + 0.0008x + 26.459; R^2^ 0.97; Min: 1333 FTU/kg; ^5^ % Albumin: y = −0.0000003x^2^ + 0.0009x + 64.127; R^2^ 0.0093; Max 1500 FTU/kg; ^6^ Shell thickness: y = −0.000003x^2^ + 0.0073x + 386; R^2^ 0.93 Max 1217 FTU/kg; ^7^ Strength: y = −0.0000002x^2^ + 0.0003x + 3.2277; R^2^ 0.98; Max 750 FTU/kg; ^8^ Yolk color: y = −0.00000008x^2^ + 0.0003x + 6.6756; R^2^ 0.81; Max: 1875 FTU/kg; ^9^ Specific gravity: y = −0.000000002x^2^ + 0.00001x + 1.0802; R^2^ 0.99; Max: 1750 FTU/kg.

**Table 3:** Morphometry of the duodenum and of the epithelium of the magnum of laying hens at 64 weeks of age fed different levels of phytase.

Treatment FTU Level		Crypt Depth (μm) ^1^	Villus Height (μm) ^2^	Villus–Crypt Ratio ^3^	Epithelium of the Magnum (μm) ^4^
0		205.48	1907.65	9.78	23.75
500		168.94	1886.33	11.85	28.27
1000		154.70	1627.82	11.85	30.27
1500		149.92	1506.95	10.63	32.28
3000		208.34	1830.22	9.19	40.60
CV (%)		25.73	17.29	32.50	25.00
*p*-values		<0.0001	<0.0001	<0.0001	<0.0001
Regression	L	0.0516	0.0223	0.0046	<0.0001
	Q	<0.0001	<0.0001	<0.0001	0.7958

Regression equation to individual variable: ^1^ Crypt depth: y = 0.00003x^2^ − 0.0752x + 203.66; R^2^ 99; Min: 1253 FTU/kg; ^2^ Villus height: y = 0.0001x^2^ − 0.486x + 1973.3; R^2^ 0.82; Min: 2430 FTU/kg; ^3^ Villus–crypt ratio: y = −0.0000008x^2^ + 0.0019x + 10.307; R^2^ 0.70; Max: 1188 FTU/kg; ^4^ Epithelium of the magnum: 0.0054x + 24.599; R^2^ 0.99.

## Data Availability

Dataset available on request from the authors.

## References

[1] Casey A., Walsh G. (2004). Identification and Characterization of a phytase of potential commercial interest. J. Biotechnol..

[2] Gautier A.E., Walk C.L., Dilger R.N. (2018). Effects of a high level of phytase on broiler performance, bone ash, phosphorus utilization, and phytate dephosphorylation to inositol. Poult. Sci..

[3] Selle P.H., Ravidran V. (2007). Microbial phytase in poultry nutrition. Anim. Feed Sci. Technol..

[4] Selle P.H., Ravidran V. (2008). Phytate-degrading enzymes in pig nutrition. Livest. Sci..

[5] Liu N., Ru Y.J., Li F.D., Wang J., Lei X. (2009). Effect of dietary phytate and phytase on proteolytic digestion and growth regulation of broilers. Arch. Anim. Nutr..

[6] Karadas F., Pirgozliev V., Pappas A.C., Acamovic T., Bedford M.R. (2010). Effects of different dietary phytase activities on the concentration of antioxidants in the liver of growing broilers. J. Anim. Physiol. Anim. Nutr..

[7] Józefiak D., Ptak A., Kaczmarek S., Maćkowiak P., Sassek M., Slominski B.A. (2010). Multi-carbohydrase and phytase supplementation improves growth performance and liver insulin receptor sensitivity in broiler chickens fed diets containing full-fat rapeseed. Poult. Sci..

[8] Ramos A.H., Santos L.M., Miglino M.A., Peres J.A., Guerra R.R. (2011). Biometria, histologia e morfometria do sistema digestório do cachorro-do-mato (Cerdocyon thous) de vida livre. Biotemas.

[9] Ishak K., Baptista A., Bianchi L., Callea F., De Groote J., Gudat F., Denk H., Desmet V., Korb G., Roderick N.M. (1995). Histological grading and staging of chronic hepatitis. J. Hepat..

[10] Agresti A. (1996). Multicategory logit models. An Introduction to Categorical Data Analysis.

[11] Marchewka J., Sztandarski P., Zdanowska-Sąsiadek Ż., Adamek-Urbańska D., Damaziak K., Wojciechowski F., Riber A.B., Gunnarsson S. (2021). Gastrointestinal Tract Morphometrics and Content of Commercial and Indigenous Chicken Breeds with Differing Ranging Profiles. Animals.

[12] Güler S., Asmaz E.D., Kayapınar N.V., İşbilir İ., Cengiz Ş.Ş., Yeşilbağ D., Şanlı A.B., Gültepe E.E. (2022). Effects of Dietary Calcium, Phosphorus and Microbial Phytase on Intestinal Morphology in Laying Hens. Turk. J. Vet. Anim. Sci..

[13] Arruda A.M.V., Fernandes R.T.V., Silva J.M., Lopes D.C. (2008). Avaliação morfo-histológica da mucosa intestinal de coelhos alimentados com diferentes níveis e fontes de fibras. Rev. Caat.

[14] Cowieson A.J., Acamovic T., Bedford M.R. (2004). The effects of phytase and phytic acid on the loss of endogenous amino acids and minerals from broiler chickens. Br. Poult. Sci..

[15] Waldroup P.W., Kersey J.H., Saleh E.A., Fritis C.A., Yan F., Stilborn H.L., Crum R.C., Raboy V. (2000). Nonphytate phosphorus requirements and phosphate excretion of broiler chicks fed diets composed of normal or high available phosphate corn with and without microbial phytase. Poult. Sci..

[16] Ravindran V., Cabahug S., Ravindran G., Selle P.H., Bryden W.L. (2000). Response of broiler chickens to microbial phytase supplementation as influenced by dietary phytic acid and non-phytate phosphorus levels. II. Effects on apparent metabolisable energy, nutrient digestibility and nutrient retention. Br. Poult. Sci..

[17] Viveros A., Brenes A., Arija I., Centeno C. (2002). Effects of microbial phytase supplementation on mineral utilization and serum enzyme activities in broiler chicks fed different levels of phosphorus. Poult. Sci..

[18] Lima M.R., Perazzo Costa F.G., Guerra R.R., Silva J.H.V., Rabello C.B.V., Miglino M.A., Lobato G.B.V., Netto S.B.S., Dantas L.S. (2013). Threonine: Lysine ratio for Japanese quail hen diets. J. Appl. Poult. Res..

[19] Johnson P.A., Reece W.O. (2006). Reprodução de Aves. Dukes, Fisiologia dos Animais Domésticos.

[20] Bunchasak C., Silapasorn T. (2005). Effects of adding methionine in low-protein diet on production performance, reproductive organs and chemical liver composition of laying hens under tropical conditions. Inter. J. Poult. Sci..

[21] Wang X., Zhu P., Sun Z., Zhang J., Sun C. (2021). Uterine Metabolomic Analysis for the Regulation of Eggshell Calcification in Chickens. Metabolites.

[22] Hamilton R.M.G. (1982). Methods and factors that affect the measurement of egg shell quality. Poult. Sci..

[23] Stadelman W.J., Coterrill P. (1995). Egg Science and Technology.

[24] Garcia E.A., Mendes A.A., Pizzolante C.C., Gonçalves H.C., Oliveira R.P., Silva M.A. (2002). Efeitos dos Níveis de Cantaxantina na Dieta sobre o Desempenho e Qualidade dos Ovos de Poedeiras Comerciais. Rev. Bras. Cien Tech. Avicola.

[25] Beeson L., Walk C.L., Bedford M.R., Olukosi O.A. (2017). Hydrolysis of phytate to its lower esters can influence the growth performance nutrient utilization of broilers with regular or doses of phytase. Poult. Sci..

[26] Dilworth L., Omoruyi L.F.O., Asemota H.N. (2005). Digestive and absorptive enzymes in rats fed phytic acid extract from sweet potato (Ipomoea batatas). Diabetol. Croat..

[27] Liu N., Ru Y.J., Li F.D., Cowieson A.J. (2008). Effect of diet containing phytate and phytase on the activity and messenger ribonucleic acid expression of carbohydrase and transporter in chickens. J. Anim. Sci..

[28] Truong H.H., Yu S., Peron A., Cadogan D.J., Khoddami A., Roberts T.H., Liu S.Y., Selle P.H. (2014). Phytase supplementation of maize-, sorghum- and wheat-based broiler diets with identified starch pasting properties influences phytate (IP6) and sodium jejunal and ileal digestibility. Anim. Feed Sci. Technol..

[29] Truong H.H., Bold R.M., Liu S.Y., Selle P.H. (2015). Standard phytase inclusion in maize-based broiler diets enhances digestibility coefficients of starch, amino acids and sodium in four small intestinal segments and digestive dynamics of starch and protein. Anim. Feed Sci. Technol..

[30] Truong H.H., Shukun Y., Moss A.F., Partridge G.G., Liu S.Y., Selle P.H. (2017). Phytase inclusions of 500 and 2000 FTU/kg in maize-based broiler diets impact on growth performance, nutrient utilization, digestive dynamics of starch, protein (N), sodium and IP6 phytate degradation in the gizzard and four small intestinal segments. Anim. Feed Sci. Technol..

